# Prevalence of treatment resistant schizophrenia according to minima TRRIP criteria in a mental health catchment area in southern Spain

**DOI:** 10.1192/j.eurpsy.2022.410

**Published:** 2022-09-01

**Authors:** L.I. Muñoz-Manchado, J.M. Pascual Paño, C. Rodríguez-Gómez, J.M. Mongil-Sanjuan, J.I. Pérez-Revuelta, R. Torrecilla-Olavarrieta, F. González-Saiz, J.M. Villagrán-Moreno

**Affiliations:** UGC North of Cadiz, Mental Health Inpatient Unit, General Hospital., Jerez de la Frontera, Spain

**Keywords:** Treatment Resistance, clozapine, schizophrénia

## Abstract

**Introduction:**

The response to antipsychotic treatment in patients with schizophrenia varies from 14 to 34% in first episodes, and from 45 to 61% in more chronic patients. Nevertheless, the concept of treatment resistant schizophrenia (TRS) is still a matter of great controversy. Recently, an international group of experts has developed the TRRIP criteria to define treatment resistant schizophrenia (TRS), including an ultra-resistance category for clozapine resistant patients. Up till now, there is a scarcity of epidemiological data of TRS with TRRIP criteria.

**Objectives:**

This study attempts to identify the population diagnosed of schizophrenia that fulfils the minima TRRIP criteria for TRS in our mental health catchment area.

**Methods:**

A descriptive and retrospective study has been developed on the patients diagnosed of schizophrenia (ICD.10, F.20) in the catchment area of the Mental Health Service at Jerez Hospital between 2018 and 2019. TRRIP criteria were applied for two independent researchers and, in case of disagreement, consensus was reached by using the LEAD procedure.

**Results:**

The total number of ICD-10 schizophrenic patients identified was 590, from a population of 456.752 in 2019. A group of these, 206 patients (35%) qualified as TRS according to the minima TRRIP criteria, 50% were positive subtype and the rest the negative one. 46.8% were treated with clozapine.

**Conclusions:**

Consensus criteria of TRS minimise the heterogeneity of epidemiological data in literature. Our data suggest a prevalence rate of TRS lower than that of similar studies. Accordingly, a comprehensive understanding of this population would undoubtedly contribute to improve preventive and therapeutic strategies.

**Disclosure:**

No significant relationships.

